# Reassessing the Larval Consumption Hypothesis in Neanderthal Diet: A Quantitative and Multi‐Proxy Evaluation

**DOI:** 10.1002/ajpa.70316

**Published:** 2026-07-16

**Authors:** José Luis Guil‐Guerrero

**Affiliations:** ^1^ Food Technology Division University of Almería Almería Spain

**Keywords:** foraging theory, insect larvae hypothesis, Neanderthal diet, stable nitrogen isotopes, trophic enrichment

## Abstract

Persistent enrichment of Neanderthal collagen δ^15^N relative to local herbivore baselines has long stimulated debate regarding Neanderthal trophic ecology. A recent proposal suggests that the consumption of fly larvae from decomposing carcasses may have contributed to this enrichment. Here, this hypothesis is evaluated using a quantitative isotopic mixing model that integrates Middle Paleolithic herbivore baselines, published larval δ^15^N data, and multiple trophic enrichment factors. Across the modeled scenarios, larvae would need to supply substantial fractions of total dietary protein to achieve the absolute target of 14‰ observed in Neanderthal collagen. Because the modeled herbivore baselines range from 4‰ to 8‰, reaching this absolute target mathematically requires spanning an isotopic gap of +6‰ to +10‰. Consequently, the required larval contributions frequently exceed 40%, and in many baseline scenarios, exceed 100% of total protein intake. Sensitivity analyzes further show that plausible variation in herbivore baselines, larval δ^15^N values, or trophic enrichment factors does not modify this outcome. These results indicate that larval intake cannot independently account for Neanderthal nitrogen isotope enrichment and, at most, would represent a minor and isotopically negligible component of dietary protein. The consistent trophic enrichment of Neanderthal collagen relative to associated herbivore baselines is therefore more parsimoniously explained by high‐trophic‐level terrestrial carnivory, potentially amplified by environmental and ecological baseline variation.

## 1 Introduction

The recent study by Beasley et al. (2025) offers a welcome new perspective on a long‐standing question in Paleolithic archeology: the origin of the persistent δ^15^N enrichment observed in Neanderthal collagen relative to co‐occurring herbivores, suggesting that they occupied a high trophic level. This pattern has conventionally been interpreted as evidence of a diet dominated by large herbivores and other high‐trophic‐level animal protein. However, alternative explanations for elevated nitrogen isotope values have also been proposed to account for this enrichment. One recent hypothesis proposes that the consumption of fly larvae developing in decomposing carcasses could contribute to elevated δ^15^N values in Neanderthal collagen. Because larvae feeding on decomposing tissues can exhibit elevated δ^15^N values relative to the original carcass tissues, their consumption could theoretically influence consumer isotope values (Beasley et al. 2025). Their contribution is timely because, despite decades of research, no single explanatory model has fully accounted for the diversity and magnitude of Neanderthal δ^15^N values across sites, regions, and time periods.

A strength of the larval consumption hypothesis is that it prompts a reconsideration of how protein sources beyond mammalian tissue may have contributed to the nitrogen isotopic signatures of Pleistocene hominins. The possibility that Neanderthals occasionally consumed larvae—whether intentionally harvested or incidentally ingested with aged meat—deserves careful evaluation. Importantly, the hypothesis expands the conceptual vocabulary available to researchers studying Paleolithic diets. It reminds us that past subsistence strategies may have included behaviors that leave little or no trace in the archeological record, and that modern ethnographic assumptions do not necessarily delineate the full breadth of hominin dietary flexibility.

At the same time, several key variables require clarification to assess the potential contribution of larvae to Neanderthal δ^15^N values. The first relates to the *quantitative dietary impact* of larval ingestion. While Beasley et al. demonstrated isotopic enrichment in larvae relative to their substrates, the extent to which this could influence human collagen values has not yet been modeled within a dietary mixing framework. The second relates to the *ecological frequency and nutritional viability* of delayed or decomposed meat consumption as part of Neanderthal subsistence. Although Neanderthals undoubtedly practiced diverse butchering, transport, and consumption strategies, the degree to which systematic meat aging and larval harvesting formed a recurrent dietary component remains uncertain. A third consideration involves the *integration of complementary isotopic proxies* beyond bulk collagen δ^15^N, which may help assess the larval hypothesis more robustly.

We note that interpretations of Neanderthal meat‐use strategies have substantially evolved. Earlier characterizations assumed that carcass processing and consumption occurred rapidly following a kill, but recent archeological research indicates a more complex picture. Cut‐mark analyzes, transport patterns, and bone‐surface modifications increasingly suggest that Neanderthal carcass management practices varied across time and space and sometimes included delayed consumption, transport of select tissues, or behaviors consistent with food storage or caching (e.g., Soulier and Morin 2016; Agam and Barkai 2018; Gaudzinski‐Windheuser, Kindler, MacDonald, et al. 2023; Gaudzinski‐Windheuser, Kindler, and Roebroeks 2023; Costamagno et al. 2006). These findings highlight the necessity of avoiding generalizations regarding Neanderthal subsistence behavior and reinforce the importance of evaluating the larval consumption hypothesis in light of this diversity.

It is also essential to acknowledge that direct archeological evidence of larval consumption would be unlikely to be preserved. The potential invisibility of this behavior in the material record reflects broader limitations inherent to Paleolithic archeology—particularly with respect to soft‐tissue and ephemeral foodstuffs—and therefore absence of evidence should not be taken as evidence of absence. Rather than centering the critique on archeological traces, the present comment focuses on the *quantitative plausibility* of the hypothesis and on identifying additional analytical strategies that could evaluate it going forward.

Here, we aim to refine and extend Beasley et al.'s proposal by integrating a two‐source isotopic mixing model to assess the dietary proportion of larvae that would be required to achieve the elevated absolute δ^15^N values (e.g., a target of 14‰) frequently observed in Neanderthal collagen.

We then outline how emerging multi‐proxy isotope approaches—including compound‐specific amino acid analysis (CSIA‐AA) and noncollagenic enamel isotopes such as zinc (δ^66^Zn) and calcium (δ^44^Ca)—may provide additional means of testing the hypothesis, even in the absence of preserved larvae. Rather than dispute the relevance of larval consumption, our goal is to delineate the boundary conditions under which larvae could materially contribute to the elevated isotopic signatures observed in Neanderthal collagen and to encourage future empirical testing.

By offering a quantitative baseline and proposing complementary analytical pathways, this comment seeks to contribute to a more nuanced understanding of the larval hypothesis and its place within the broader spectrum of Neanderthal dietary behavior. We regard this as an opportunity for constructive dialog: strengthening the original proposal through modeling, situating it within current archeological evidence, and pointing toward future research that may determine when, where, and to what extent larval consumption could have influenced Paleolithic human isotope values.

Unless otherwise specified, references to Neanderthal δ^15^N values in this study refer to trophic enrichment relative to co‐occurring herbivore baselines.

## 2 Materials and Methods

### 2.1 Isotopic Baselines for Herbivore Tissues

Herbivore δ^15^N values representative of Middle Paleolithic European ecosystems were compiled from published faunal isotope datasets. These values serve as local isotopic baselines against which Neanderthal collagen δ^15^N values are interpreted. Because nitrogen isotope values vary substantially between regions and time periods due to environmental and ecological factors, expressing consumer values relative to their associated herbivore baseline provides a more robust framework for cross‐site comparison.

Nitrogen isotope enrichment was expressed as the isotopic difference between Neanderthal collagen and associated herbivore baselines:

$$\Delta^{15}N=\delta^{15}N_{\mathrm{Neanderthal}}-\delta^{15}N_{\mathrm{herbivore}}\;\;$$

Empirical values for this enrichment derived from published datasets (Table S1) indicate typical Δ^15^N values of approximately +4‰ to +7‰. However, to standardize the quantitative isotopic mixing model, we established a fixed absolute Neanderthal collagen target of 14‰—representing the upper range of these observed values—against which to evaluate the required larval contribution.

### 2.2 Larval δ^15^N Inputs and Isotopic Parameter

Published δ^15^N values for insect larvae associated with decomposing animal tissues were compiled from experimental and ecological studies. These studies show that larval tissues can become enriched in ^15^N relative to the decomposing substrate as a result of nitrogen recycling and trophic processing during decomposition.

In several experimental contexts, larval δ^15^N values exceed those of the underlying muscle substrate by more than +15‰. However, these values represent substrate‐to‐larva enrichment and do not translate directly into equivalent changes in human collagen values. The isotopic impact of larval consumption depends on the absolute δ^15^N values of the larvae, the proportion of dietary protein derived from them, and the trophic enrichment factor between diet and consumer tissues.

To capture this uncertainty, the modeling framework incorporated a broad range of plausible larval δ^15^N values, including highly enriched scenarios consistent with the ranges reported in experimental decomposition studies.

### 2.3 Trophic Enrichment Factor (TEF) Scenarios

Trophic enrichment factors (TEF) between dietary protein and consumer bone collagen were modeled using values of +3‰, +4‰, and + 5‰. This range encompasses the values commonly reported for mammalian carnivores and Pleistocene hominins in stable isotope studies (e.g., Bocherens and Drucker 2003; O'Connell et al. 2012), while allowing for physiological variability related to protein routing and dietary composition. It is important to distinguish TEF from Δ^15^N. TEF represents the physiological isotopic enrichment between dietary protein and consumer collagen, whereas Δ^15^N represents the observed isotopic difference between Neanderthal collagen and the associated herbivore baseline (Section 2.1). Consequently, Δ^15^N reflects both physiological trofic enrichment and the isotopic composition of the consumed dietary protein, whereas TEF accounts only for the collagen‐diet fractionation. The two quantities are therefore related but not equivalent.

Diets composed primarily of high‐quality animal protein typically require less metabolic reworking and may result in slightly lower isotopic fractionation between diet and consumer tissues. Because Neanderthal diets are widely interpreted as heavily reliant on animal protein, exploring this TEF range allows the model to capture plausible variability in trophic enrichment under different physiological conditions.

### 2.4 Isotopic Mixing Model

To evaluate the potential contribution of insect larvae to Neanderthal nitrogen isotope values, a simple linear isotopic mixing model was implemented describing the proportional contribution of larval and herbivore protein sources to the nitrogen isotopic composition of consumer collagen.

In its absolute form, the model can be expressed as follows:

$$\delta^{15}N_{\text{target}}=f_{\text{larvae}}\;\delta^{15}N_{\mathrm{larvae}}+\left(1-f_{\text{larvae}}\right)\;\delta^{15}N_{\mathrm{herbivore}}+\mathrm{TEF}$$

where δ^15^N_target_ represents the fixed Neanderthal collagen δ^15^N value target. δ^15^N_larvae_ represents the δ^15^N value of larval protein. δ^15^N_herbivore_ represents the δ^15^N value of herbivore muscle protein. *f*_larvae_ represents the proportion of dietary protein derived from larvae. TEF represents the trophic enrichment factor between dietary protein and consumer collagen.

Because ecological baselines vary among archeological sites and time periods, model outputs are interpreted relative to the asociated herbivore baseline using the Δ^15^N framework defined in Section 2.1. This relative enrichment provides a standarized measure of trophic spacing that facilitates comparison among archeological contexts while accounting for regional variation in baseline nitrogen isotope values.

To determine the required larval proportion, the baseline absolute mixing equation was rearranged to solve for the dietary fraction of protein derived from insect larvae (*f*_larvae_):

$$f_{\text{larvae}}=\frac{\left(\delta^{15}N_{target}-\mathrm{TEF}\right)‐\delta^{15}N_{\text{herbivore}}}{\delta^{15}N_{\text{larvae}}-\delta^{15}N_{\text{herbivore}}}$$

where *f*_larvae_ represents the fraction of dietary protein derived from larvae. δ^15^N_target_ represents the fixed Neanderthal collagen δ^15^N value target (e.g., 14‰). δ^15^N_larvae_ represents the δ^15^N value of larval protein. δ^15^N_herbivore _represents the δ^15^N value of herbivore muscle protein. TEF represents the trophic enrichment factor between dietary protein and consumer collagen. For interpretation, model outputs are expressed within the Δ^15^N framework introduced in Section 2.1, where Δ^15^N denotes the isotopic difference between Neanderthal collagen and the associated herbivore baseline.

**FIGURE 1:**
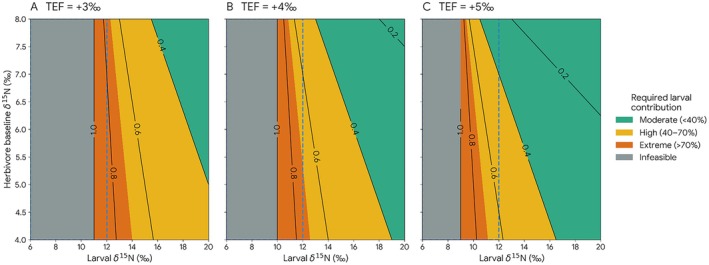
Nitrogen isotope mixing model evaluating the contribution of insect larval protein to highly elevated Neanderthal collagen δ^15^N values. Predicted combinations of larval and herbivore δ^15^N values capable of producing a representative Neanderthal collagen isotopic composition (absolute δ^15^N = 14‰) are shown for three trophic enrichment factor (TEF) scenarios between dietary protein and consumer collagen: (A) +3‰, (B) +4‰, and (C) +5‰. Contour lines indicate the proportion of dietary protein derived from larvae (*f*ₗₐᵣᵥₐₑ = 0.2–1.0) required to achieve the target collagen value, while colored regions categorize the magnitude of this contribution: Moderate (< 40%, green), high (40%–70%, yellow), extreme (> 70%, orange), and isotopically infeasible (*f*ₗₐᵣᵥₐₑ > 1.0 or < 0, gray). The blue dashed rectangle delineates the ecologically realistic parameter space (larval δ^15^N = 6‰–12‰; herbivore δ^15^N = 4‰–8‰) based on available archeological and ecological observations. Within this realistic parameter space, moderate larval contributions (< 40%) are absent, and most parameter combinations require high (40%–70%), extreme (> 70%), or isotopically infeasible larval contributions. Interpreted within a Δ^15^N framework, achieving an absolute Neanderthal collagen value of 14‰ against herbivore baselines of 4‰–8‰ requires a trophic enrichment of approximately +6‰ to +10‰. Under these conditions, larval consumption alone would need to constitute a disproportionately large fraction of total dietary protein to reproduce the observed isotopic signal. The model therefore supports the interpretation that insect larvae may have contributed to Neanderthal diets but are unlikely to represent the primary driver of elevated Neanderthal δ^15^N values when considered in isolation.

Parameter combinations yielding mathematical impossibilities (e.g., *f*_larvae_ < 0 or *f*_larvae_ > 1) were considered isotopically infeasible and are represented by the dark gray regions in the resulting contour plots (Figure 1). The model is intended as a heuristic exploration of isotopic plausibility rather than a quantitative dietary reconstruction and therefore evaluates the parameter space under which larval consumption could theoretically account for the highly elevated absolute δ^15^N targets observed in Neanderthals.

### 2.5 Model Exploration and Sensitivity Analysis

Model simulations evaluated the combinations of absolute larval δ^15^N values, trophic enrichment factors (TEFs), and dietary proportions required to achieve a fixed, elevated target Neanderthal collagen value of 14‰, based on upper‐range absolute values from published archeological datasets (Table S1). Across the modeled herbivore baselines (4‰–8‰), achieving this absolute target mathematically corresponds to bridging a relative isotopic gap (Δ^15^N) of +6‰ to +10‰. Sensitivity analyzes (Table S2) were then conducted to examine how systematic variation across the entire parameter space—including absolute larval δ^15^N values, herbivore baselines, and TEF parameters—influences the proportion of larval‐derived protein required to reach typical Neanderthal collagen targets ranging from 12‰ to 15‰.

Contour plots were generated to visualize the combinations of absolute larval δ^15^N values, herbivore baselines, and dietary proportions required to reach the elevated absolute δ^15^N targets (e.g., 14‰) typically observed in Neanderthal collagen under different TEF scenarios. These plots illustrate the strict mathematical boundary conditions under which larval consumption could materially influence these high isotopic signatures.

All calculations were implemented using a deterministic mixing model. The algorithm used to generate the model and the R script used to produce the contour plots are provided in the Supporting Information (Code S1).

## 3 Results

### 3.1 Behavior of the Isotopic Mixing Model

The isotopic mixing model shows that insect larvae, even when assigned high δ^15^N values, exert only limited influence on consumer collagen δ^15^N unless they constitute a very large fraction of total dietary protein. For combinations of herbivore muscle δ^15^N (4‰–8‰) and larval δ^15^N (6‰–12‰) consistent with Middle Paleolithic contexts (indicated by the dashed rectangle in Figure 1), the model indicates that achieving the 14‰ Neanderthal collagen target requires larval protein contributions that generally range from 25% to over 100%, heavily dependent on the baseline and specific TEF applied.

These patterns are clearly visualized in Figure 1, where the contour fields for TEF = +3‰, +4‰, and + 5‰ show large areas in which the required larval fraction (*f*_larvae_) is either > 1.0 (impossible) or < 0 (biologically meaningless). While broad bands of intermediate values do produce mathematically feasible mixing scenarios (0.0 to 1.0), reaching the target frequently requires dietary compositions dominated by larvae at levels incompatible with known hominin feeding ecology.

### 3.2 Effects of Trophic Enrichment Factor (TEF)

Varying the trophic enrichment factor from +3‰ to +5‰ produces predictable but limited shifts in the required larval contribution. Higher TEFs reduce the larval proportions needed to raise consumer δ^15^N; however, even under the most favorable scenario (TEF = +5‰), achieving the target Neanderthal collagen value still requires larvae to supply at least ~25% of total dietary protein within the realistic ecological parameter space. TEF variation, therefore, alters the contour structure but does not meaningfully change the overall conclusion that larvae cannot be the primary driver of the elevated absolute Neanderthal δ^15^N values.

### 3.3 Sensitivity Analysis

The sensitivity analysis (Table S2) evaluated the fraction of all modeled parameter combinations (herbivore δ^15^N = 4.0‰–8.0‰; larval δ^15^N = 6.0‰–12.0‰; evaluated at a 0.1‰ grid step) that yield biologically feasible required larval dietary protein fractions (*f*_*L*_) at or below specific thresholds to achieve typical target Neanderthal collagen values of 12‰, 14‰, and 15‰. Across all TEF scenarios (+3‰ to +5‰) and target values, the parameter space allowing for biologically realistic larval consumption remains strictly limited. For the primary target of 14‰, 0.0% of the parameter space allows for a larval contribution of ≤ 20% across all TEF scenarios, and feasible overall dietary solutions (*f*_*L*_ ≤ 1.00) represent at most 50.8% of the modeled conditions (at TEF = +5‰). Even for a lower target of 12‰ under the most favorable conditions (TEF = +5‰), only 14.8% of the parameter space yields a larval dietary proportion of ≤ 20%. These results demonstrate that, despite broad variation in isotopic parameters, larval‐derived protein would need to constitute a substantial proportion of total dietary protein to appreciably elevate consumer δ^15^N values, reinforcing the fundamental interpretation that larval intake alone cannot account for the observed enrichments.

### 3.4 Overall Model Outcome

Together, the contour plots and sensitivity analyzes demonstrate that insect larvae cannot independently or substantially account for the highly elevated absolute δ^15^N values observed in Neanderthal collagen. They may have been consumed opportunistically, but their isotopic influence is negligible compared with herbivore‐derived protein and trophic enrichment. While the model does not independently verify the specific sources of high‐trophic terrestrial carnivory, it demonstrates that larvae—if present—constituted at most a minor dietary component.

## 4 Discussion

### 4.1 Archeological and Ethnohistorical Context of Larval Consumption

The proposition that Neanderthals may have occasionally consumed fly larvae as part of decomposing meat expands the range of dietary pathways considered in Paleolithic subsistence research. Ethnographic and ethnohistorical records from diverse human populations show that larvae and other insects have been consumed opportunistically—sometimes as fallback foods, sometimes deliberately—and often with little or no specialized harvesting technology. In this respect, Beasley et al. (2025) correctly highlight that larval consumption could leave few, if any, durable traces in the archeological record.

The lack of direct material evidence should therefore not be overinterpreted. As emphasized by Wallach (2019), caution is warranted when drawing inferences from absence, particularly for activities that involve soft tissues, ephemeral foodstuffs, or behaviors unlikely to produce persistent residues. This applies especially to practices such as the ingestion of larvae from naturally decomposing carcasses, for which archeological signatures would be minimal.

For these reasons, the present analysis does not question the behavioral plausibility of occasional larval consumption by Neanderthals. The ingestion of larvae—whether incidentally during butchery or intentionally as a nutrient‐rich resource—fits within the broader spectrum of foraging behaviors documented among recent hunter‐gatherers. However, the key question is not whether such behaviors could have occurred, but whether they could have done so with sufficient frequency and magnitude to exert a measurable effect on Neanderthal collagen δ^15^N values at the population level.

The following sections, therefore, shift from qualitative considerations to quantitative assessment, focusing on the isotopic implications of larval intake within realistic ecological bounds. This allows the larval hypothesis to be evaluated on the basis of explicit mass‐balance constraints rather than on assumptions about archeological preservation.

### 4.2 Implications of the Mixing Model for Dietary Contribution

The isotopic mixing model developed here provides a quantitative framework for estimating the proportion of dietary protein from fly larvae required to reach the elevated absolute δ^15^N values commonly observed in Neanderthals (e.g., a target of 14‰). Because Middle Paleolithic large herbivore baselines typically range from 4‰ to 8‰, reaching this absolute target mathematically necessitates bridging an isotopic gap of +6‰ to +10‰, with occasional higher offsets. Across all trophic enrichment scenarios tested (TEF = +3 − +5‰), the model indicates that substantial proportions of dietary protein derived from larvae would be required to reproduce the empirically observed Δ^15^N enrichment.

Even under the most favorable assumptions within the realistic parameter space—high larval δ^15^N values (12‰), a high herbivore baseline (8‰), and a maximum standard TEF of +5‰—the proportion of larval‐derived protein required remains considerable at roughly 25%. Under lower TEF values or lower baselines, the estimated contribution increases rapidly to levels (> 40% to > 100%) that are ecologically and behaviorally implausible given what is known about Middle Paleolithic subsistence, the energetics of hominin foraging, and the ephemeral, limited nature of larval biomass on decomposing carcasses. These findings do not exclude the possibility that Neanderthals consumed larvae. Still, they indicate that such consumption would have a negligible isotopic impact unless it were both frequent and constituted a major component of dietary protein.

The results of the sensitivity analysis presented in Table S2 reinforce this interpretation. Evaluation across the entire modeled parameter space demonstrates that these boundary constraints are robust; under no modeled scenario can realistic larval consumption (≤ 20%) bridge the isotopic gap to the 14‰ target. This confirms the overarching conclusion that only high and sustained larval intake—far beyond what opportunistic or incidental consumption would provide—could generate the trophic enrichment documented between Neanderthal collagen and local herbivore baselines. By quantifying these thresholds, the model clarifies the boundary conditions of the Beasley et al. (2025) proposal and identifies the circumstances under which a larval pathway could influence isotopic signatures, as well as the conditions under which its contribution becomes negligible.

It is important to clarify that some reported larval δ^15^N values—such as those cited by Beasley et al. (2025)—show enrichments exceeding +15‰ relative to muscle tissue in specific experimental or ecological contexts. These values represent substrate‐to‐larva differences and may reflect intensive nitrogen recycling during decomposition. However, such enrichments do not translate directly into equivalent shifts in human collagen, which depends on the absolute δ^15^N of the consumed larvae, the proportion of total dietary protein they represent, and the trophic enrichment factor between diet and consumer tissue. To address this uncertainty, the present modeling incorporates a broad range of plausible larval δ^15^N values, including highly enriched scenarios. Even under these expanded conditions, larvae must constitute a disproportionately large fraction of total dietary protein to reproduce the trophic enrichment typically observed in Neanderthal collagen relative to herbivore baselines. Furthermore, even if Neanderthals selectively and exclusively targeted maximally enriched larvae (+15‰ relative to the carcass, yielding absolute larval values approaching 20‰), mass‐balance requirements dictate that these larvae would still need to constitute a disproportionately large percentage of their total annual protein intake (approx. 15%–25%) to independently drive the bulk collagen signal. Sustaining such a volume year‐round via opportunistic harvesting from ephemeral carcasses remains ecologically improbable. The central constraint therefore arises from proportional dietary requirements rather than from assumptions about low larval enrichment per se.

### 4.3 Neanderthal Carcass Use and the Feasibility of Delayed Meat Consumption

Assessing the dietary relevance of larval consumption also requires consideration of Neanderthal carcass processing strategies and the temporal dynamics of meat use. Earlier interpretations often suggested that Neanderthals rapidly consumed fresh meat following a kill, but extensive zooarcheological evidence accumulated in recent decades indicates a far more variable behavioral repertoire. Cut‐mark distributions, skeletal element transport patterns, and the spatial organization of faunal remains collectively demonstrate that carcass exploitation differed across regions, seasons, and cultural phases. Several studies explicitly documented delayed consumption, the transport of selected high‐value tissues, and practices interpreted as forms of meat storage or caching (Soulier and Morin 2016; Costamagno et al. 2006; Agam and Barkai 2018; Gaudzinski‐Windheuser, Kindler, MacDonald, et al. 2023; Gaudzinski‐Windheuser, Kindler, and Roebroeks 2023).

This behavioral heterogeneity cautions against applying a single universal model of carcass use. It also implies that conditions suitable for larval development—such as partial exposure of carcass tissues for extended periods—could plausibly have occurred in certain contexts. The processing of very large animals or multiple prey within a short interval may have exceeded immediate consumption capacity, potentially allowing natural insect colonization of some tissues. Ethnographic and ethological analyzes likewise show that temporary caching or short‐term storage of meat can serve as an adaptive risk‐buffering strategy under specific ecological or social conditions (Dally et al. 2006; Stopp 2002).

However, for larval consumption to exert a meaningful effect on Neanderthal trophic enrichment relative to local herbivore baselines, such behaviors would need to be recurrent and systematic across the individuals and sites displaying elevated isotopic signatures. Occasional or situational delays in meat use—while plausible—would produce only marginal isotopic effects and are unlikely to account for the consistently high δ^15^N values documented at several Middle Pala eolithic sites. The combined archeological and ecological evidence therefore suggests that, although delayed meat use and incidental larval ingestion form part of the range of possible Neanderthal behaviors, the conditions required for larvae to become a major dietary protein source appear limited. In the absence of sustained or widespread reliance on decomposed meat enriched with larvae, the larval pathway is best interpreted as supplementary or context‐specific behavior rather than a primary driver of elevated Neanderthal trophic enrichment above associated herbivore baselines.

Although consumption of insect larvae could theoretically contribute to elevated nitrogen isotope values, the modeling results indicate that very high dietary reliance on larvae would be required to reach the absolute 14‰ target (which mathematically necessitates bridging a massive +6‰ to +10‰ isotopic gap from the local herbivore baselines), suggesting that larval consumption alone is unlikely to account for the consistently elevated δ^15^N values observed in Neanderthal collagen.

### 4.4 Complementary Explanations for Elevated Neanderthal δ^15^N

Persistent δ^15^N enrichment of Neanderthal collagen relative to co‐occurring herbivores has long been interpreted as indicative of a high trophic position, consistent with substantial reliance on large herbivore muscle tissue (Bocherens 2009; Bocherens and Drucker 2003). To fully contextualize the highly elevated isotopic signatures of Neanderthals, it is necessary to compare them not only against herbivore baselines but also against contemporaneous apex predators. Recent comprehensive faunal datasets, such as those compiled for Late Pleistocene ecosystems by Smith et al. (2024), demonstrate that apex carnivores like gray wolves ( *Canis lupus* ) and spotted hyenas ( *Crocuta crocuta* ) typically yield mean δ^15^N values of approximately +8.4‰ and + 7.9‰, respectively. This reflects an expected trophic enrichment (Δ^15^N) of roughly +3‰ to +4‰ above the local herbivore baseline (+4.0‰ to +5.1‰). In stark contrast, Neanderthal collagen values frequently target absolute values of 12‰ to 14‰ within similar ecological contexts (Table S1). This results in a massive Δ^15^N offset of +6‰ to +10‰ relative to herbivores, positioning Neanderthals significantly higher than even the true apex carnivores of their ecosystems. This differential enrichment clearly illustrates the “trophic anomaly” of Neanderthals. Reaching such an extreme isotopic threshold necessitates either the targeted consumption of hyper‐enriched megafauna (e.g., mammoths), severe physiological/environmental baseline shifts, or both. Furthermore, the magnitude of this gap mathematically reinforces the conclusion that the consumption of insect larvae—even highly enriched specimens—would require entirely implausible dietary proportions to independently drive Neanderthal collagen signals so far beyond the apex carnivore baseline. At several sites, access to large‐bodied mammals—such as straight‐tusked elephants or mammoths—has been proposed as an additional driver of high consumer δ^15^N, whether through the consumption of top‐level prey or through specialized hunting and processing strategies (Gaudzinski‐Windheuser, Kindler, MacDonald, et al. 2023; Gaudzinski‐Windheuser, Kindler, and Roebroeks 2023). Other explanatory mechanisms have also been documented. These include prolonged breastfeeding effects in subadult individuals, aridity‐driven enrichment of terrestrial food webs, and physiological factors affecting nitrogen routing, metabolic stress, or tissue turnover rates (O'Connell et al. 2012; Jaouen et al. 2022). In more localized cases, freshwater resource exploitation has been considered, particularly in regions where aquatic ecosystems exhibit enriched baseline δ^15^N values.

Situating larval consumption within this broader interpretive framework is essential. Consistent with the perspective outlined by Beasley et al. (2025), the ingestion of larvae is best regarded as *one possible contributor* to elevated Neanderthal δ^15^N values rather than a primary or universal mechanism. The modeling results presented here reinforce this position: larvae could contribute to overall nitrogen intake under particular ecological or behavioral circumstances but would do so alongside other well‐established dietary and environmental factors. The intention of this comment is therefore not to reject the larval pathway outright, but to delineate more clearly the scale of its potential influence relative to other drivers and to integrate it into a multifactorial model of Neanderthal nitrogen isotope enrichment.

As frequently proposed by Bocherens and colleagues, the targeted hunting of megafauna, particularly mammoths, remains a leading hypothesis for these extreme trophic elevations in certain regions. However, it must be acknowledged that no single explanatory model—whether mammoth hunting, aridity, physiological stress, or larval consumption—has yet proven universally applicable. The ultimate cause of these elevated values likely involves a complex, regionally variable mosaic of behaviors and environments that we are still working to fully explain.

### 4.5 Multi‐Proxy Isotopic Approaches to Testing the Hypothesis

Bulk collagen δ^15^N measurements alone are insufficient to disentangle the relative contributions of different protein sources, nor can they differentiate reliably between terrestrial, aquatic, or insect‐derived pathways. For this reason, multi‐proxy isotopic approaches offer the most promising avenues for evaluating the larval consumption hypothesis. Compound‐specific amino acid analysis (CSIA‐AA) is particularly valuable in this regard. By comparing δ^15^N values of source amino acids such as phenylalanine—reflecting baseline ecosystem signatures—with trophic amino acids such as glutamate, CSIA‐AA can distinguish trophic‐level effects from environmental influences (Chikaraishi et al. 2009, 2014). Crucially for the larval hypothesis, because fly larvae on carrion consume highly degraded, microbially active substrates, their amino acid signatures (and the subsequent routing of those amino acids into a hominin consumer) may exhibit distinct detrital or microbial isotopic spacing compared to the direct consumption of fresh mammalian herbivore muscle. In contexts where larvae are not preserved archeologically, modern insect CSIA‐AA datasets provide realistic isotopic parameters that can be incorporated into future modeling efforts, allowing researchers to assess whether larval intake was required—or even compatible—with the detrital and trophic δ^15^N signatures observed in Neanderthal collagen.

Isotopic systems preserved in tooth enamel offer additional, independent constraints. Unlike collagen, enamel retains signals in elements such as zinc (δ^66^Zn) and calcium (δ^44^Ca), which respond differently to trophic level and dietary composition and are not affected by soft‐tissue preservation biases. The notably low δ^66^Zn value reported for the Gabasa Neanderthal enamel is consistent with a diet dominated by high‐trophic‐level terrestrial animal protein and is difficult to reconcile with a substantial contribution of insect‐based protein sources (Jaouen et al. 2022). When combined with bulk δ^15^N and amino‐acid‐specific analyzes, these enamel‐based proxies form a coherent multi‐proxy framework that can more robustly evaluate the isotopic plausibility of the larval hypothesis and situate it within a broader understanding of Neanderthal dietary ecology.

### 4.6 Strengths and Limitations of This Study

The approach presented here provides a quantitative refinement of the larval consumption hypothesis by defining the boundary conditions under which larvae could meaningfully influence Neanderthal collagen δ^15^N values. One of the principal strengths of this study is the explicit modeling of the dietary proportions required for larval intake to shift consumer isotope values, thereby clarifying the circumstances in which the hypothesis becomes isotopically plausible. Incorporating multiple trophic enrichment factors (TEFs) and a realistic range of source δ^15^N values further underscores how sensitive isotopic interpretations are to variation in these parameters, a point often acknowledged but rarely quantified in debates surrounding Neanderthal diets.

Nevertheless, several limitations must be recognized. The model simplifies nitrogen metabolism by treating dietary protein sources as isotopically homogeneous and linearly additive, an approach that necessarily overlooks the complexity of metabolic routing, tissue‐specific fractionation, and physiological responses to diet. Variability in larval δ^15^N values—including species‐level differences, substrate heterogeneity, and environmental influences—introduces additional uncertainties that cannot be resolved without broader empirical datasets. The model also does not incorporate potential interactions between trophic and physiological processes that might attenuate or amplify isotopic signals in ways difficult to predict. Accordingly, the framework developed here should be interpreted as exploratory: it is intended to guide future, more detailed investigations rather than to serve as a comprehensive reconstruction of Neanderthal dietary behavior.

### 4.7 Synthesis and Outlook

Taken together, the archeological, isotopic, and behavioral lines of evidence suggest that larval consumption remains a plausible, though likely secondary, component of Neanderthal subsistence. The quantitative modeling presented here indicates that larvae alone are unlikely to account for the highly elevated absolute δ^15^N values typical of many Neanderthal individuals, unless consumed at frequencies and intensities for which current archeological and ecological evidence is limited. Nevertheless, the hypothesis merits continued exploration. The framework outlined in this comment—drawing on bulk collagen, compound‐specific amino acid analysis, and enamel‐based isotope systems—provides an avenue for evaluating the larval pathway within a broader and more integrated dietary context.

Advancing this discussion will depend on empirical datasets capable of distinguishing larval contributions from other trophic processes, particularly in regions where environmental baselines or prey ecologies vary substantially. Applying multi‐proxy isotopic techniques to Neanderthal remains from diverse temporal and environmental settings will be essential for determining when, where, and to what extent larvae might have contributed to hominin diets. In this sense, the larval hypothesis serves not as a restrictive alternative to established interpretations, but as a catalyst for expanding the analytical toolkit used to reconstruct Neanderthal subsistence. By encouraging attention to overlooked dietary pathways and new isotopic approaches, it broadens, rather than narrows, the possibilities for understanding the complexity and flexibility of Paleolithic lifeways.

## 5 Conclusion

The larval consumption hypothesis proposed by Beasley et al. (2025) represents an innovative contribution to ongoing discussions of the elevated absolute δ^15^N values observed in Neanderthals. The aim of this comment has not been to dismiss the possibility of larval intake, but to refine its interpretive scope by assessing the dietary proportion required for larvae to exert a measurable isotopic effect. The mixing model results show that, under reasonable assumptions, larvae would need to constitute a substantial share of total dietary protein to reach these highly elevated absolute δ^15^N targets independently of other dietary factors. This finding suggests that larval consumption is more convincingly interpreted as a complementary rather than a primary dietary driver.

Considering the hypothesis within a broader interpretive landscape underscores the importance of multiple, potentially interacting mechanisms—including consumption of high‐trophic‐level herbivore tissues, environmental and physiological influences, and regionally specific factors such as mammoth exploitation or freshwater resource use. Moving forward, the most promising avenue for evaluating the larval pathway lies in multi‐proxy isotopic approaches. Combining bulk collagen data with compound‐specific amino acid analysis and enamel‐based isotope systems provides a robust framework for determining whether—and under what conditions—larval consumption could have contributed to Neanderthal nitrogen signals.

By reframing the question in this way, the present comment encourages continued exploration of underexamined dietary behaviors while promoting empirical tests capable of distinguishing among competing explanations. The goal is not to curtail debate, but to sharpen it: to move from theoretical possibility toward demonstrable plausibility through evidence capable of untangling the respective contributions of behavior, environment, and physiology to Neanderthal isotopic signatures.

## Author Contributions

**José Luis Guil‐Guerrero:** conceptualization, investigation, funding acquisition, writing – original draft, writing – review and editing, visualization, validation, methodology, software, formal analysis, project administration, data curation, supervision, resources.

## Conflicts of Interest

The author declares no conflicts of interest.

## Supporting information


**Code S1.** Deterministic isotopic mixing model for evaluating the contribution of insect larval protein to Neanderthal δ^15^N values.

**Table S1:** Published Neanderthal collagen δ^15^N values and associated faunal baselines used to define the trophic enrichment range evaluated in the mixing model.

**Table S2:** Sensitivity analysis of the isotopic mixing model showing how variation in larval δ^15^N values, herbivore baseline δ^15^N, and trophic enrichment factors (TEF) affects the estimated dietary proportion of larvae required to reproduce specified Neanderthal collagen δ^15^N values.

## Data Availability

The data that supports the findings of this study are available in the Supporting Information of this article.
