# Supplementary material for: Reassessing the Larval Consumption Hypothesis in Neanderthal Diet: A Quantitative and Multi‐Proxy Evaluation
Source: Am J Biol Anthropol. 2026 Jul 16;190(3):e70316. doi: 10.1002/ajpa.70316 (PMC13376460; doi:10.1002/ajpa.70316)
Supplement: Supplementary file 1 — Code S1. Deterministic isotopic mixing model for evaluating the contribution of insect larval protein to Neanderthal δ15N values. [file AJPA-190-e70316-s001.pdf]

Supplementary Code S1. Deterministic Isotopic Mixing Model for Evaluating the Contribution of Insect Larval Protein to Neanderthal δ^{15}N Values

```
#
================================================================================
=

# Supplementary Code S1. Deterministic Isotopic Mixing Model for Evaluating

# the Contribution of Insect Larval Protein to Neanderthal δ15N Values

# Figure 1: Nitrogen Isotope Mixing Model - Final Publication Version

#
================================================================================
=


# Load necessary libraries

library(ggplot2)

library(dplyr)

library(metR)

library(grid)


# ---------------------------------------------------------------------------

# 1. Define Parameter Space & Calculate Model

# ---------------------------------------------------------------------------

herbivore  <- seq(4, 8, length.out = 500)

larvae     <- seq(6, 20, length.out = 500)

tef_values <- c(3, 4, 5)

c_val      <- 14  # Target Neanderthal collagen absolute δ15N (‰)


# Create full parameter grid

model_data <- expand.grid(

  herbivore = herbivore,
```

```
  larvae    = larvae,
  tef       = tef_values
) %>%
  mutate(
    denom = larvae - herbivore,

    # Stable calculation avoiding division by zero
    fL = ifelse(abs(denom) < .Machine$double.eps^0.5,
                NA,
                ((c_val - tef) - herbivore) / denom),

    # Classification of larval contribution
    zone = case_when(
      is.na(fL)              ~ "Infeasible",
      fL < 0                 ~ "Infeasible",
      fL >= 0.0 & fL <= 0.4    ~ "Moderate (<40%)",
      fL > 0.4 & fL <= 0.7     ~ "High (40–70%)",
      fL > 0.7 & fL <= 1.0     ~ "Extreme (>70%)",
      fL > 1.0               ~ "Infeasible"
    ),

    # Lock legend order
    zone = factor(zone, levels = c(
      "Moderate (<40%)",
      "High (40–70%)",
      "Extreme (>70%)",
      "Infeasible"
```

```r
    ))
  )

# ---------------------------------------------------------------------------
# 2. Build the Figure
# ---------------------------------------------------------------------------
final_figure <- ggplot(model_data, aes(x = larvae, y = herbivore)) +

  # Background zones
  geom_raster(aes(fill = zone), alpha = 0.85) +

  scale_fill_manual(
    values = c(
      "Moderate (<40%)" = "#1b9e77",
      "High (40–70%)"   = "#e6ab02",
      "Extreme (>70%)"  = "#d95f02",
      "Infeasible"      = "#636e72"
    ),
    # Matches the safe text formatting used in the manuscript and caption
    name = "Larval dietary contribution (f_larvae)",
    drop = FALSE
  ) +

  # Contour lines
  geom_contour(
    aes(z = fL),
    breaks = c(0.2, 0.4, 0.6, 0.8, 1.0),
```

```
  colour = "black",
  linewidth = 0.8
) +

# Centered contour labels (critical for final match)
geom_text_contour(
  aes(z = fL),
  breaks = c(0.2, 0.4, 0.6, 0.8, 1.0),
  label.placer = label_placer_fraction(0.5),
  size = 5.5,
  fontface = "bold",
  color = "black",
  skip = 0
) +

# Ecologically realistic parameter space
annotate("rect",
        xmin = 6, xmax = 12,
        ymin = 4, ymax = 8,
        linetype = "dashed",
        linewidth = 1.2,
        colour = "#2980b9",
        fill = NA) +

# Faceting by TEF
facet_wrap(~tef, labeller = labeller(
  tef = c(
```

```
    "3" = "A  TEF = +3‰",
    "4" = "B  TEF = +4‰",
    "5" = "C  TEF = +5‰"
  )
)) +

# Axis labels with correct Greek notation
labs(
  x = expression(bold("Larval " * delta^{15} * "N (‰)")),
  y = expression(bold("Herbivore baseline " * delta^{15} * "N (‰)"))
) +

# Fixed axis limits
coord_cartesian(xlim = c(6, 20), ylim = c(4, 8), expand = FALSE) +

# Publication-ready theme
theme_classic(base_size = 18) +
theme(
  strip.text       = element_text(face = "bold", size = 20, hjust = 0),
  strip.background = element_blank(),
  axis.title       = element_text(face = "bold", size = 18),
  axis.text        = element_text(size = 14, color = "black"),
  legend.title     = element_text(face = "bold", size = 16),
  legend.text      = element_text(size = 15),
  legend.position  = "right",
  panel.border     = element_rect(colour = "black", fill = NA, linewidth = 1),
  panel.spacing    = unit(1.5, "lines")
```

```
  )

# ------------------------------------------------------------------------------
# 3. Export High-Resolution Figures
# ------------------------------------------------------------------------------

# TIFF (journal standard)
ggsave("Figure1_Final.tiff",
       plot = final_figure,
       width = 16,
       height = 7,
       dpi = 600,
       compression = "lzw",
       units = "in")

# PDF (vector format)
ggsave("Figure1_Final.pdf",
       plot = final_figure,
       width = 16,
       height = 7,
       units = "in")

print("Figure successfully generated and saved.")
```
