# Supplementary material for: Reassessing the Larval Consumption Hypothesis in Neanderthal Diet: A Quantitative and Multi‐Proxy Evaluation
Source: Am J Biol Anthropol. 2026 Jul 16;190(3):e70316. doi: 10.1002/ajpa.70316 (PMC13376460; doi:10.1002/ajpa.70316)
Supplement: Supplementary file 2 — Table S1: Published Neanderthal collagen δ15N values and associated faunal baselines used to define the trophic enrichment range evaluated in the mixing model. [file AJPA-190-e70316-s003.pdf]

Supplementary Table S1. Published Neanderthal collagen δ^{15}N values and associated faunal baselines used to define the trophic enrichment range evaluated in the mixing model.

Site	Region	Approx. Age (ka)	Neanderthal δ^{15}N (‰)	Carnivore δ^{15}N (‰)	Associated Herbivore δ^{15}N (‰)	Neanderthal Δ^{15}N (‰)	Carnivore Δ^{15}N (‰)	Reference
Scladina Cave Layer 4A/4B	Belgium	~80–127	10.9	8.9	7.2	+3.7	+1.7	Bocherens (1999)
Vindija Cave	Croatia	~28.5	~10.5	~9.7	~5.3	+5.2	+4.4	Richards et al. (2000)
Les Pradelles	France	~36	~11.4	~8.3	~4.7	~6.7[a]	~3.6[a]	Bocherens et al. (2005); Fizet et al. (1995); Bocherens (2009)
Les Rochers-de-Villeneuve	France	35.2	~11.6	Hyena: ~8.5-9.5	~4.5	~+7.1[b]	~4-5[b]	Beauval et al. (2006)
Jonzac	France	40–55	~10.9	Hyena: 8.6	~6.1	+3.8	+2.5	Richards et al. (2008)
Okladnikov Cave Altai	Siberia	~30–44	~13.5	~9.8[c]	~7.0[c]	~+6.5	~+2.8	Dobrovolskaya and Tiunov (2013)
Spy Cave	Belgium	~40.6–44.2	~11.1	10.5	~5.2[d]	~+5.9	~+5.3	Naito et al. (2016); Wißing et al. (2016)
Goyet	Belgium	~40–45	~11.32	10.5[e]	~5.2[e]	~+6.12[e]	~+5.3[e]	Wißing et al. (2019)
Les Cottés	France	~40–45	~13.9	11.3	~7.7	~+6.2	~+3.6	Jaouen et al. (2019)
Chagyrskaya Cave, the Altai Mountains	Siberia	~59	~13.8	~8.6[f]	~6.8[f]	~+7.0	~+1.8	Salazar García et al. (2021)

Generic Notes:

- Bulk Neanderthal values and herbivore baselines vary intra-site; reported ranges are approximate means based on published figures from site or nearby fauna where available.
- "Associated Herbivore δ^{15}N" is based on published large herbivore values from the same site or contemporaneous fauna from nearby contexts.
- Δ^{15}N is calculated as δ^{15}N$_{Neandertal}$ – δ^{15}N$_{herbivore}$ and represents the trophic offset used to define target enrichment in modelling.
- Values represent either site means or representative specimens depending on the level of resolution reported in the original publication.

Specific Notes:

[a] The **δ^{15}N** trophic paradox at Les Pradelles—where Neanderthals exhibit an apparent **Δ^{15}N** offset of **≈ +6.7‰** above the general herbivore baseline (~4.7‰)—is driven by prey selection skews and hyper-carnivorous protein metabolism rather than anomalous mammalian fractionation. Multi-source

mixing models demonstrate that Neanderthals preferentially targeted high-nitrogen megaherbivores like woolly mammoths (*Mammuthus primigenius*, ~7.5‰–8.5‰); accounting for this hidden baseline aligns the actual trophic enrichment factor with the standard mammalian offset of +3.5‰ to +5.0‰ (Bocherens et al., 2005). This dietary signature was further elevated by physiological routing, as near-exclusive protein consumption accelerates urea clearance, which selectively excretes ^{14}N and concentrates heavy $\mathbf{^{15}N}$ into bone collagen (Bocherens, 2009; Fizet et al., 1995). Consequently, compound-specific amino acid analysis confirms that these high bulk values reflect top-predator trophic status rather than mathematical artifacts (Jaouen et al., 2019). To standardize the values in the table, the mathematically obtained values have been included directly, as in the other evaluated sites.

[b] The apparent trophic inflation paradox at Les Rochers-de-Villeneuve ($\mathbf{\Delta^{15}N}$_Neanderthal-Fauna **≈ +7.1‰**) mirrors the mathematical discrepancy observed at Les Pradelles, where direct subtraction of the average local herbivore baseline yields a biologically impossible mammalian fractionation step. Rather than indicating an anomalous metabolic process, this highly elevated signature represents a combination of open-steppe foraging skews and hyper-carnivorous protein routing. Because the immediate faunal assemblage recovered from this hyena-den context is structurally biased toward medium-sized ungulates, the standard linear baseline calculation lacks representation of local high-nitrogen megaherbivores (such as woolly mammoths), which Neanderthals preferentially targeted elsewhere in the landscape. Accounting for this unquantified megafaunal baseline, combined with the metabolic concentration of heavy $\mathbf{^{15}N}$ caused by accelerated urea clearance on a near-exclusive meat diet, aligns the actual trophic enrichment factor with the standard mammalian offset of +3.5‰ to +5.0‰, confirming the individual's top-predator ecological status (Beauval et al., 2006).

[c] For Okladnikov Cave, herbivore and carnivore baselines are represented by the analysed bison and wolf specimens reported by Dobrovolskaya and Tiunov (2013), the only herbivore and carnivore reported.

[d] Approximate herbivore baseline obtained from the central tendency of the principal herbivore guilds discussed in the paper (reindeer, bovids, horse, rhinoceros, mammoth). This value is **calculated**, not explicitly reported by the authors.

[e] Herbivore and carnivore baseline values are derived from the associated Belgian faunal isotopic framework established for Spy/Goyet by Wißing et al. (2016).

References

Beauval, C., Lacrampe-Cuyaubère, F., Maureille, B., & Trinkaus, E. (2006). Direct radiocarbon dating and stable isotopes of the Neandertal femur from Les Rochers-de-Villeneuve (Lussac-les-Châteaux, Vienne). Bulletins et Mémoires de la Société d’Anthropologie de Paris, 18(1-2), 35–42.

Bocherens, H. (2009). Neanderthal dietary habits: review of the isotopic evidence. In: The Evolution of Hominin Diets. Springer, Dordrecht.

Bocherens, H., Billiou, D., Mariotti, A., Patou-Mathis, M., Otte, M., Bonjean, D., & Toussaint, M. (1999). Palaeoenvironmental and palaeodietary implications of isotopic biogeochemistry of last interglacial Neanderthal and mammal bones in Scladina Cave (Belgium). Journal of archaeological science, 26(6), 599-607.

Bocherens, H., Drucker, D. G., Billiou, D., Patou-Mathis, M., & Vandermeersch, B. (2005). Isotopic evidence for diet and subsistence pattern of the Saint-Césaire I Neanderthal: review and use of a multi-source mixing model. Journal of human evolution, 49(1), 71-87.

Dobrovolskaya, M. V., & Tiunov, A. V. (2013). The Neanderthals of Okladnikov Cave Altai: Environment and diet based on isotopic analysis. Archaeology, Ethnology and Anthropology of Eurasia, 41(1), 78-88.

Fizet, M., Mariotti, A., Bocherens, H., et al. (1995). Effect of diet, physiology and climate on carbon and nitrogen stable isotopes of collagen in a late Pleistocene anthropic palaeoecosystem: Marillac, Charente, France. Journal of Archaeological Science, 22(1), 67-79.

Jaouen, K., Richards, M. P., Le Cabec, A., Welker, F., Rendu, W., Hublin, J. J., ... & Talamo, S. (2019). Exceptionally high δ15N values in collagen single amino acids confirm Neandertals as high-trophic level carnivores. Proceedings of the National Academy of Sciences, 116(11), 4928-4933.

Kuzmin, Y. V., Bondarev, A. A., Kosintsev, P. A., & Zazovskaya, E. P. (2021). The Paleolithic diet of Siberia and Eastern Europe: evidence based on stable isotopes (δ13C and δ15N) in hominin and animal bone collagen. *Archaeological and Anthropological Sciences*, *13*(10), 179.

Richards, M. P., Pettitt, P. B., Trinkaus, E., Smith, F. H., Paunović, M., & Karavanić, I. (2000). Neanderthal diet at Vindija and Neanderthal predation: the evidence from stable isotopes. Proceedings of the National Academy of Sciences, 97(13), 7663-7666.

Richards, M. P., Taylor, G., Steele, T., McPherron, S. P., Soressi, M., Jaubert, J., ... & Hublin, J. J. (2008). Isotopic dietary analysis of a Neanderthal and associated fauna from the site of Jonzac (Charente-Maritime), France. Journal of Human Evolution, 55(1), 179-185.

Salazar-García, D. C., Power, R. C., Rudaya, N., Kolobova, K., Markin, S., Krivoshapkin, A., ... & Viola, B. (2021). Dietary evidence from Central Asian Neanderthals: A combined isotope and plant microremains approach at Chagyrskaya Cave (Altai, Russia). Journal of Human Evolution, 156, 102985.

Wißing, C., Rougier, H., Crevecoeur, I., Germonpré, M., Naito, Y.I., Semal, P., & Bocherens, H. (2016). *Isotopic evidence for dietary ecology of late Neandertals in North-Western Europe*. Quaternary International 411, 327–345.

Wißing, C., Rougier, H., Baumann, C., Comeyne, A., Crevecoeur, I., Drucker, D. G., ... & Bocherens, H. (2019). Stable isotopes reveal patterns of diet and mobility in the last Neandertals and first modern humans in Europe. Scientific reports, 9(1), 4433.
