# Supplementary material for: Reassessing the Larval Consumption Hypothesis in Neanderthal Diet: A Quantitative and Multi‐Proxy Evaluation
Source: Am J Biol Anthropol. 2026 Jul 16;190(3):e70316. doi: 10.1002/ajpa.70316 (PMC13376460; doi:10.1002/ajpa.70316)
Supplement: Supplementary file 3 — Table S2: Sensitivity analysis of the isotopic mixing model showing how variation in larval δ15N values, herbivore baseline δ15N, and trophic enrichment factors (TEF) affects the estimated dietary proportion of larvae required to reproduce specified Neanderthal collagen δ15N values. [file AJPA-190-e70316-s002.pdf]

Supplementary Table S2. Sensitivity analysis of the isotopic mixing model showing how variation in larval δ^{15}N values, herbivore baseline δ^{15}N, and trophic enrichment factors (TEF) affects the estimated dietary proportion of larvae required to reproduce specified Neanderthal collagen δ^{15}N values.

TEF (‰)	Target collagen δ^{15}N (‰)	$f_L \leq 0.20$	$f_L \leq 0.50$	$f_L \leq 1.00$
3	12	0.0	9.2	50.8
3	14	0.0	0.0	18.0
3	15	0.0	0.0	1.6
4	12	10.0	35.2	68.0
4	14	0.0	0.0	34.4
4	15	0.0	0.0	18.0
5	12	14.8	47.2	68.0
5	14	0.0	9.2	50.8
5	15	0.0	0.0	34.4

Fraction of modelled herbivore-larvae δ^{15}N parameter combinations (herbivore δ^{15}N = 4.0–8.0‰; larval δ^{15}N = 6.0–12.0‰; evaluated at a grid step of 0.1‰) that yield biologically feasible required larval dietary protein fractions ($f_{larvae} \geq 0$) at or below specified thresholds (≤0.20, ≤0.50, and ≤1.00) to achieve the indicated consumer target collagen δ^{15}N values. Percentages represent the fraction of the total parameter space (n = 2,501 combinations). Values are rounded to one decimal place. Percentages falling outside the ≤1.00 threshold indicate that the target δ^{15}N is mathematically unattainable under a feasible two-source mixing scenario within the defined parameters. Results demonstrate that, across all tested scenarios, larval-derived protein would need to constitute a substantial proportion of total dietary protein to appreciably elevate consumer δ^{15}N values to the typical Neanderthal targets.
